# Anticholinergic burden and frailty in older inpatients: insights from analysis of admission and discharge medicines using four anticholinergic scales

**DOI:** 10.1186/s12877-024-05394-3

**Published:** 2024-12-20

**Authors:** Mohammed Adem Mohammed, Amy Hai Yan Chan, Nasir Wabe, Ayesha Ali, Louis Harris, Sianne West, Rhea Colaabavala, Justine Aw, Jeff Harrison

**Affiliations:** 1 School of Pharmacy, Faculty of Medical and Health Sciences, The University of Auckland, Auckland, New Zealand; 2 Australian Institute of Health Innovation, Macquarie University, Sydney, Australia

**Keywords:** Anticholinergic burden, Frailty, Older adults, Older inpatients, Acute setting, Deprescribing

## Abstract

**Background:**

Exposure to high anticholinergic burden is associated with adverse outcomes in older adults. Older adults with frailty have greater vulnerability to adverse anticholinergic effects. There is limited data on anticholinergic burden in hospitalised older adults with frailty particularly, in New Zealand. This study aimed to (i) examine exposure to anticholinergic medicines in older inpatients using multiple scales, and (ii) describe the association of patient factors such as frailty with anticholinergic exposure.

**Methods:**

We reviewed admission and discharge medicines of 222 older patients (≥ 65 years) in a New Zealand hospital. Sociodemographic, diagnostic and medication data were collected from electronic health records. Anticholinergic burden was quantified using the Anticholinergic Burden Classification (ABC), Anticholinergic Cognitive Burden Scale (ACB), Anticholinergic Risk Scale (ARS), and Drug Burden Index (DBI). Frailty was assessed using frailty index (FI) and the Hospital Frailty Risk score (HFRS); higher scores indicate higher frailty. Multivariable logistic regression analysis was used to determine patient factors associated with anticholinergic burden.

**Results:**

Depending on the scale used, the mean anticholinergic burden ranged from 0.65 to 1.83 on admission and 0.59 to 1.40 at discharge, with 32–74% of the patients on admission and 25–65% at discharge prescribed at least one anticholinergic medicine. About 1 in 3 patients had high anticholinergic burden on admission and discharge. On admission, being frail (adjusted odds ratio [AOR] 5.16, 95% confidence interval [95% CI] 1.57, 16.97), having history of readmission (AOR 4.96, CI 1.58, 15.59), and higher number of medicines [AOR range 1.18 CI 1.10, 1.26 (ARS scale) to 1.25 CI 1.15, 1.36 (DBI scale)] were associated with higher odds of anticholinergic exposure. At discharge, pre-frail (DBI scale: AOR = 6.58, CI 1.71–25.32) and frail patients (ACB scale: AOR = 5.73, CI 1.66, 19.70) and those with higher number of medicines [AOR range 1.18 CI 1.09, 1.29 (ARS scale) to 1.33 CI 1.20, 1.49 (DBI scale)] had higher odds of anticholinergic exposure.

**Conclusion:**

A reduction in the anticholinergic burden from admission to discharge was observed in the study population yet, one-third of the study cohort were discharged with high anticholinergic medicines. Enhancing hospital prescribers’ and pharmacists’ awareness about anticholinergic burden and targeted interventions such as in-hospital deprescribing are needed to reduce high anticholinergic exposure in acute setting.

**Supplementary Information:**

The online version contains supplementary material available at 10.1186/s12877-024-05394-3.

## Background

Medicines with anticholinergic effects are commonly used in older adults for the management of various long-term conditions. Evidence shows between 20% and 50% of older adults are prescribed at least one medicine with anticholinergic effect [1]. The cumulative effect of concurrent use of one or more medicines with anticholinergic properties is known as anticholinergic burden [2]. A high anticholinergic burden is a reversible risk factor for a range of adverse health outcomes including cognitive decline, falls, fracture, and hospitalisation in older adults [3, 4]. Older adults have increased susceptibility to adverse anticholinergic effects due to age-related changes in physiologic function altering pharmacokinetics and pharmacodynamics of drugs [5]. Furthermore, having multimorbidity, polypharmacy and frailty increase vulnerability to adverse anticholinergic outcomes [6]. Despite the risks of adverse anticholinergic health outcomes, the prevalence of anticholinergic use in older adults continues to increase [7, 8]. The growing trend in the anticholinergic burden has become a global concern and thus, reducing exposure to anticholinergics is a priority to optimise medicines use and outcomes in older adults.

Several tools have been developed to measure the cumulative effects of anticholinergic medicines [9–11] and assist health care providers in optimising the use of these medicines. Scales such as the Drug Burden Index (DBI) [12], Anticholinergic Risk Scale (ARS) [13], Anticholinergic Cognitive Burden Scale (ACB) [14] and Anticholinergic Burden Classification (ABC) [15] have been widely validated and used in clinical research. However, existing scales differ in their underlying assumptions for quantifying anticholinergic burden, list of medicines, and weighting assigned in rating the medicines. In addition, there is limited data comparing the performance of existing scales in measuring cumulative anticholinergic burden in relation to clinical outcomes. Currently, no single anticholinergic scale is universally accepted gold standard measure [16] and thus, a combination of more than one scale may help in providing better insights into anticholinergic exposure in the studied population.

In New Zealand and globally, research into anticholinergic burden in older adults have predominantly focused on primary healthcare or long-term care facilities [17–20]. There is limited data on anticholinergic exposure in hospitalised older adults, particularly in those with frailty to inform risk stratification and patient prioritisation for in-hospital medicines review. In this study, we aimed to examine anticholinergic prescribing in older inpatients using multiple scales and describe the association of patient factors such as frailty status with anticholinergic exposure in a New Zealand hospital.

## Methods

### Study design and setting

This was a single centre, retrospective evaluation of electronic medical records of older patients recruited from Auckland city hospital general medicine and geriatric services. These patients were enrolled in the medicines burden and attitude towards deprescribing study between February 2020 and March 2022 [21]. Patients were included in the study if they were 65 years and older, had at least one long-term condition and one prescription medicine on admission, were not cognitively impaired, and were able to provide written consent.

### Data collection

We reviewed electronic medical records of enrolled patients and extracted relevant data on sociodemographic (e.g. age, sex, ethnicity, socioeconomic deprivation), and clinical (e.g. comorbidities, history of readmission) and medication data. The New Zealand deprivation index (Dep2013) was used to evaluate socioeconomic deprivation, and the scores were categorised as least deprived (scores 1–3), moderately deprived (scores 4–7), and most deprived (scores 8–10). Medical conditions were coded using the International Classification of Diseases 10th Revision-Australian Modification (ICD-10- AM). Diagnostic data were used to assess the burden of comorbidities using the Charlson comorbidity index (CCI) [22].

### Assessment of frailty

The Hospital frailty risk score (HFRS) [23] and Frailty index (FI) [24] were used to assess frailty status (See supplementary file). These measures assess frailty based on the number of accumulated deficits which can be quantified using ICD-10-AM and other relevant information on the patient’s record at the time of index admission. Based on their HFRS score, patients are stratified as no risk (0), low risk (1–5), intermediate risk (5–15), and high risk (> 15), a higher score indicating a higher risk of frailty for a patient [23]. There were no patients in the ‘no risk’ and ‘high-risk’ category in our study and thus, HFRS was dichotomised into either low or intermediate risk. The FI was calculated by summing the number of deficits in the FI variables and dividing by the total number of possible deficits. The index ranges from 0 to 1 and based on their FI scores, patients are categorised as non-frail (0–0.25), pre-frail (0.26–0.35) and frail (0.36–1) [25].

### Assessment of anticholinergic medication exposure

Detailed information about medications such as name, dose, dose frequency, route of administration, and indication was collected for each admission and discharge medication. Exposure to anticholinergic medications on admission and at discharge was evaluated using the Anticholinergic Cognitive Burden Scale (ACB) [14], Anticholinergic Burden Classification (ABC) [15], Anticholinergic Risk Scale (ARS) [13] and the Drug Burden Index (DBI) [12]. The ACB, ABC, and ARS scales classify each medicine on a scale of 0 to 3, with 0 indicating no anticholinergic property and 3 indicating strong anticholinergic effect [11]. The DBI scale evaluates exposure to anticholinergic and sedative medications, and the total DBI is the cumulative score of each medication, where 0 represents no burden, a score > 0 but < 1 represents a low burden and a score ≥ 1 is high burden. In this study, after quantifying patient level anticholinergic burden on admission and at discharge, the scores were categorised as 0 (no exposure) and > 0 (exposure to anticholinergic medicines). Scores > 0 were further categorised into low to moderate and high anticholinergic burden using the cut-offs proposed in each scale.

### Statistical analysis

The characteristics of the study participants were summarised using descriptive statistics. Continuous variables were reported using mean and standard deviation (SD), or median and interquartile range (IQR) as appropriate. Categorical variables were reported using frequencies and percentages. The Wilcoxon Singed-Rank test [26] was used to compare anticholinergic burden score on admission and discharge for each scale. Multivariable logistic regression analyses were conducted to assess factors associated with anticholinergic burden on admission and at discharge quantified by each anticholinergic scale, with adjustment for sociodemographic (e.g. age, sex, ethnicity and socioeconomic deprivation), clinical (e.g. comorbidity, number of medicines, frailty, history of falls and readmission) covariates. We calculated unadjusted and adjusted Odds Ratios (AOR) with 95% confidence intervals (CI). We excluded patients who died during hospitalisation from the analysis at discharge. In all cases, significance was set at a *p*-value less than 0.05. Analyses were performed using SPSS software, version 28.

## Results

### Sociodemographic and clinical characteristics of the study population

A total of 222 patients were included in the study of which, seven died during hospitalisation. The mean age was 81.9 (SD 8.2), and 63% were female. The majority of the cohort was of European ethnicity (84.7%), and 18% were living in the most deprived areas. The average number of medicines on admission and at discharge were 10.6 (SD 5.1) and 9.6 (SD 4.5), respectively. The majority had polypharmacy (≥ 5 medications) on admission (89.1%) and at discharge (90.1%). Almost 1 in 4 patients had a history of readmission 28 days prior to index admission, 44.1% had a history of falls, and 48.6% had an extended length of stay (> 20 days). The mean hospital frailty risk and FI scores were 4 (SD 3) and 0.4 (SD 0.2), respectively. Most patients were frail (76.6%) as per FI and were in low risk (63.5%) category according to the hospital frailty score. The average Charlson comorbidity index score was 1.61(SD 1.70) (Table 1).

**Table 1:** Sociodemographic and clinical characteristics of study population (*N* = 222)

Age *in years*, mean (SD)	81.9 (± 8.2)
Sex female, n (%)	141 (63.5%)
Ethnicity n (%)	
European	188 (84.7%)
Asian	17 (7.7%)
Pacifica	11 (5.0%)
Māori	6 (2.7%)
Socioeconomic deprivation	
Least deprived, n (%)	84 (37.8%)
Moderately deprived, n (%)	98 (44.1%)
Most deprived, n (%)	40 (18.0%)
Number of medicines on admission, mean (SD)	10.6 (± 5.1)
Polypharmacy, n (%)	199 (89.6%)
Anticholinergic burden on admission	
ARS, mean (SD)	0.86 (1.6)
ABC, mean (SD)	1.37 (1.9)
ACB, mean (SD)	1.83 (1.8)
DBI, mean (SD)	0.65 (0.8)
Number of medicines at discharge, mean (SD)	9.6 (4.5)
Polypharmacy at discharge, n (%)	200 (93.0%)
Anticholinergic burden at discharge	
ARS, mean (SD)	0.59 (1.3)
ABC, mean (SD)	1.05 (1.6)
ACB, mean (SD)	1.40 (1.5)
DBI, mean (SD)	0.59 (0.7)
Length of stay in days, median (IQR)	20.92 (17.9)
History of falls n (%)	98 (44.1%)
History of readmission n (%)	54 (24.3%)
Hospital frailty score, mean (SD)	4.0 (3.0)
Low risk n (%)	141 (63.5%)
Intermediate risk n (%)	81 (36.5%)
Frailty index mean, (SD)	0.4 (0.2)
Frail, n (%)	170 (76.6%)
Pre-Frail, n (%)	28 (12.6%)
Non-Frail, n (%)	24 (10.8%)
Charlson comorbidity index mean, (SD)	1.61 (± 1.7)

*ARS* = Anticholinergic Risk Scale, *ABC* = Anticholinergic Burden, Classification, *ACB* = Anticholinergic Cognitive Burden, *DBI* = Drug Burden Index, *SD* = Standard deviation, *IQR* = Interquartile Range, *LOS* = Length of Stay

### Exposure to anticholinergic medicines

**Fig. 1:**
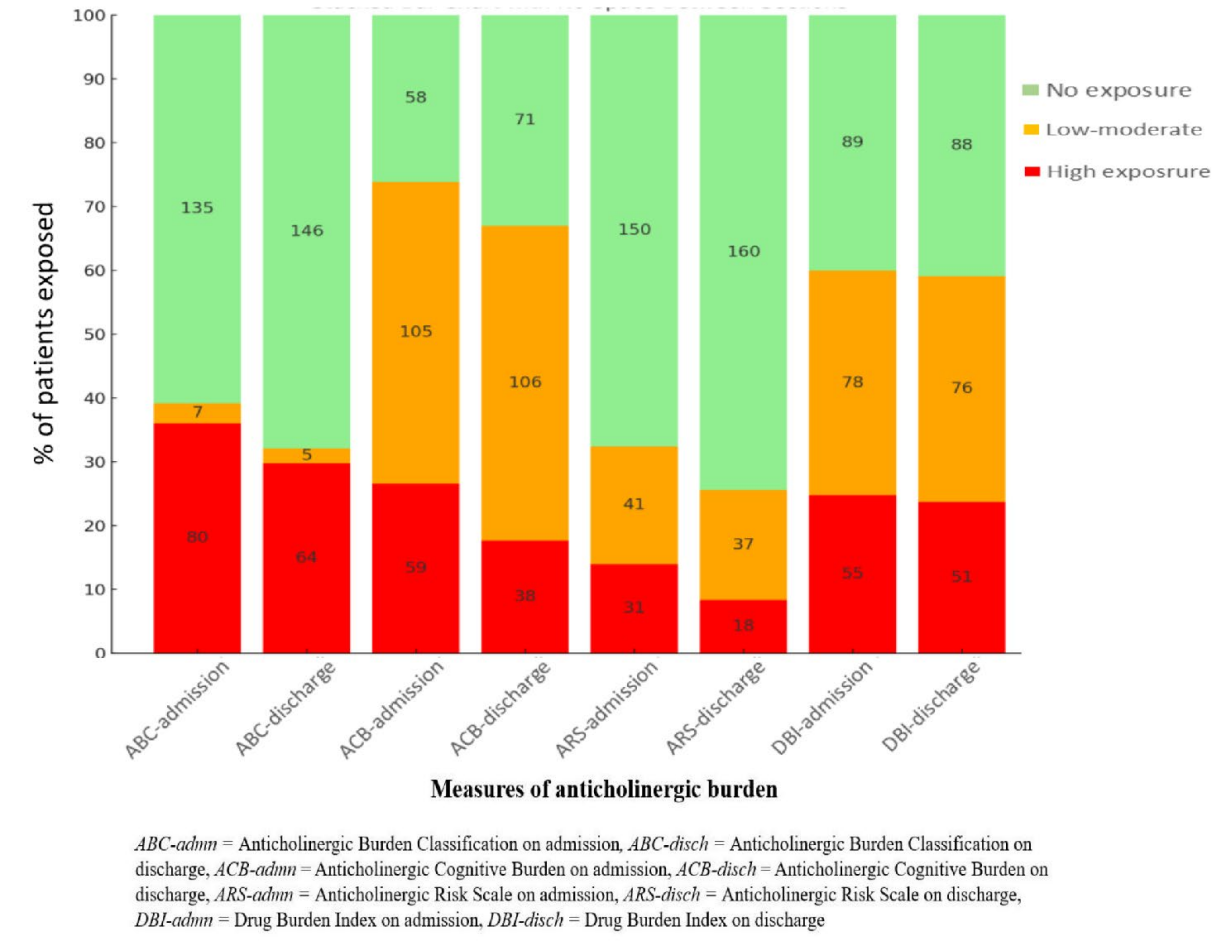
Exposure to anticholinergic medicines on admission and discharge

Exposure to anticholinergics on admission and discharge is shown in Fig. 1. On admission, 73.9%, 59.9%, 39.2% and 32.4% of the patients had exposure to at least one anticholinergic medicine (i.e. score > 0) according to the ACB, DBI, ABC and ARS scales, respectively. A ‘high’ anticholinergic burden on admission was observed in 36%, 26.6%, 24.8% and 14% of the patients according to the ABC, ACB, DBI and ARS scales, respectively. At discharge, 66.9%, 59.1%, 32.1% and 25.6% had at least one anticholinergic prescribed as per the ACB, DBI, ABC and ARS scores, respectively. A ‘high’ anticholinergic burden was observed in 29.8%, 23.7%, 17.7%, and 8.4% of patients based on the ABC, DBI, ACB and ARS scores, respectively.

Further analysis of exposure to anticholinergics by frailty showed that 39% (ACB scale) to 44.4% (ARS scale) of the patients with intermediate frailty risk, and 77.8% (ARS scale) to 86.2% (ABC scale) of frail patients had exposure to at least one anticholinergic medicine on admission. At discharge, 40.9% (DBI scale) to 56.4% (ARS scale) patients with intermediate frailty risk and 75.6% (DBI scale) to 87% (ABC scale) of frail patients had exposure to at least one anticholinergic medicine. When stratified by the level of exposure, 12.4%, 25.9%, 26.5% and 40% of patients with frailty had high anticholinergic exposure on admission as per ARS, DBI, ACB and ABC scale, respectively. Likewise, 7.9%, 18.3%, 25.6% and 34.8% of frail patients had high anticholinergic exposure at discharge as per ARS, ACB, DBI and ABC scale, respectively.

**Fig. 2:**
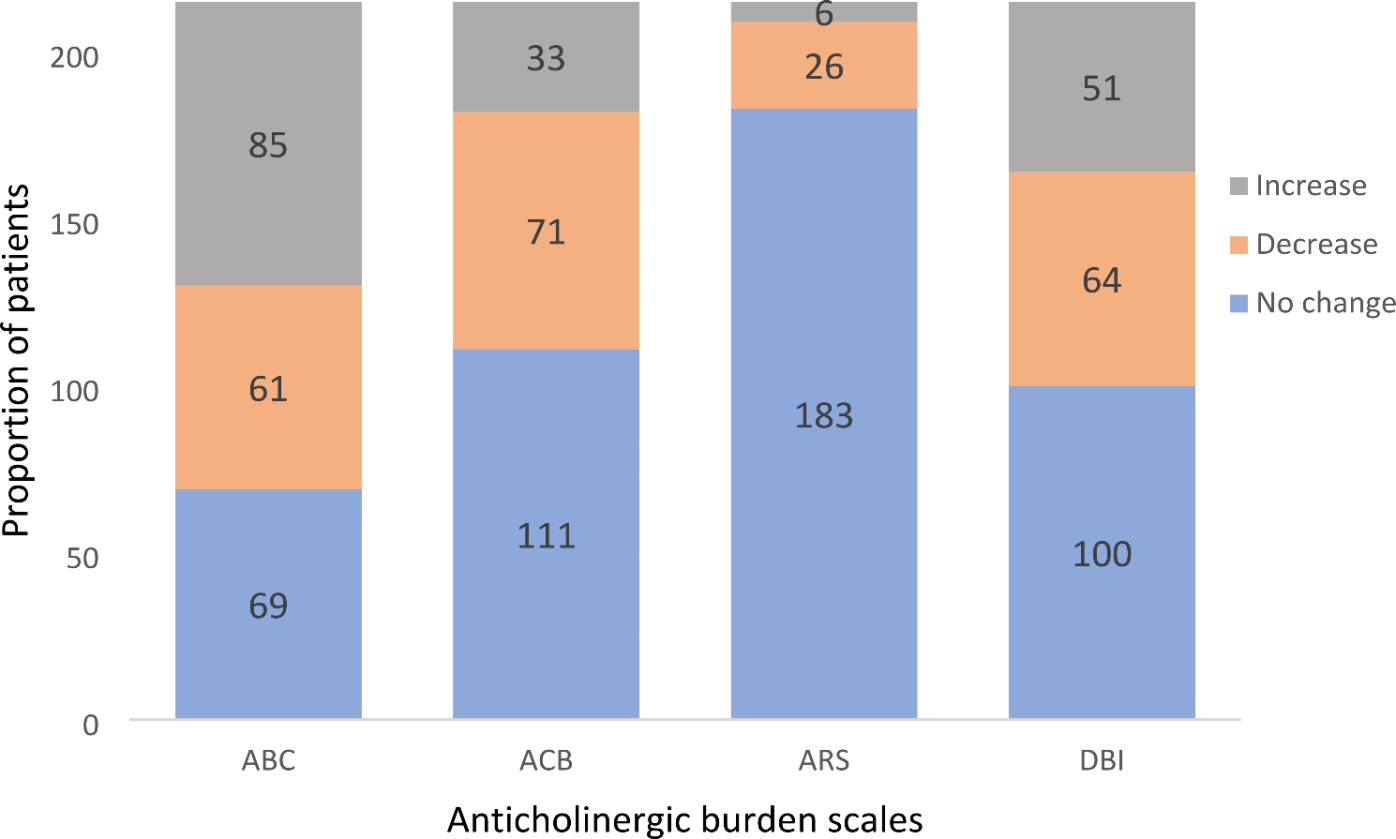
Proportion of patients with increased, decreased, and unchanged anticholinergic medicines exposure at discharge

There was a significant reduction in the median anticholinergic burden score from admission to discharge across the scales except in the DBI [ACB scale *P* < 0.001; ABC scale *P* = 0.02; ARS scale *P* < 0.001; DBI scale *P* = 0.22]. In 33% (*n* = 71 ACB scale), 29.8% (*n* = 64 DBI scale), 28.4% (*n* = 61 ABC scale), 12.1% (*n* = 26 ARS scale) of the patients, there was a reduction in the anticholinergic score from admission to discharge. However, majority of the patients had no change in their anticholinergic burden score from admission to discharge (Fig. 2).

### Factors associated with exposure to anticholinergic medicines on admission and discharge

The results of logistic regression examining sociodemographic and clinical characteristics associated with exposure to anticholinergics on admission and at discharge are shown in Tables 2 and 3. On admission, the odds of exposure to anticholinergics was higher with higher frailty score (ACB scale: AOR = 5.16, CI 1.57, 16.97), 28-day readmission history (ACB scale: AOR = 4.96, 95% CI 1.58, 15.59), and increasing number of medicines (ABC scale: AOR = 1.19, CI 1.11, 1.35; ACB scale: AOR = 1.23, CI 1.15, 1.42, ARS: AOR = 1.18, CI 1.10, 1.26, DBI scale: AOR = 1.25, CI 1.15, 1.36).

**Table 2:** Factors associated with exposure to anticholinergics on admission (*N* = 222)

Variables	Exposure to anticholinergics on Admission			
	ACB scale AOR (95% CI)	ABC scale AOR (95% CI)	ARS scale AOR (95% CI)	DBI scale AOR (95% CI)
Age in years*	1.00 *(0.95–1.05)*	1.01 *(0.97–1.05)*	0.96 *(0.92–1.01)*	0.97 *(0.93–1.02)*
Sex *(ref male)*	0.73 *(0.33–1.59)*	0.95 *(0.49–1.84)*	1.55 *(0.76–3.18)*	0.95 *(0.48–1.86)*
Ethnicity (ref NZ European)				
Asian	4.01 *(0.68–23.75)*	1.45 *(0.47–4.49)*	0.56 *(0.17–1.93)*	0.32 *(0.10–1.05)*
Pacific	0.91 *(0.18–4.55)*	0.68 *(0.14–3.27)*	0.21 *(0.04–1.28)*	0.28 *(0.06–1.29)*
Māori	0.51 *(0.07–3.94)*	1.75 *(0.27–11.20)*	0.63 *(0.08–5.05)*	0.33 *(0.04–2.47)*
Number of medicines*	**1.23** ***(1.15–1.42)***	**1.19** ***(1.11–1.28)***	**1.18** ***(1.10–1.26)***	**1.25** ***(1.15–1.36)***
Deprivation Index *(ref least deprived)*				
Moderately deprived	1.72 *(0.75–3.96)*	1.03 *(0.52–2.06)*	1.05 *(0.52–2.16)*	1.33 *(0.65–2.72)*
Most deprived	0.90 *(0.33–2.43)*	1.34 *(0.55–3.30)*	0.69 *(0.26–1.83)*	0.53 *(0.21–1.34)*
CCI	1.02 *(0.81–1.29)*	1.08 *(0.90–1.30)*	0.76 *(0.61–0.96)*	0.78 *(0.63–0.97)*
Frailty Index *(ref non-frail)*				
*Pre-frail*	1.86 *(0.44–7.87)*	0.37 *(0.08–1.77)*	0.84 *(0.20–3.49)*	3.63 *(0.88–15.11)*
*Frail*	**5.16** ***(1.57–16.97)***	1.37 *(0.40–4.65)*	1.00 *(0.31–3.28)*	1.92 *(0.63–5.87)*
HFRS *(ref low risk)*	0.81 *(0.34–1.90)*	0.960 *(0.48–1.91)*	1.88 *(0.91–3.99)*	1.71 *(0.82–3.55)*
Readmission history *(ref none)*	**4.96** ***(1.58–15.59)***	1.68 *(0.82–3.48)*	0.68 *(0.32–1.48)*	1.26 *(0.58–2.78)*
History of falls *(ref none)*	0.93 *(0.43–2.02)*	0.88 *(0.45–1.71)*	0.59 *(0.29–1.19)*	1.36 *(0.69–2.70)*

Bolded values = statistically significant (*p* < 0.05) higher odds of anticholinergic exposure; *AOR* = Adjusted Odds Ratio, *CI* = confidence interval, *ACB* = Anticholinergic Cognitive Burden, *ABC* = Anticholinergic Burden Classification, *ARS* = Anticholinergic Risk Scale, *DBI* = Drug Burden Index, ‘*ref’ =* Reference Group, *= continuous variable

At discharge, pre-frail (DBI scale: AOR = 6.58, CI 1.71, 25.32) and frail patients (ACB scale: AOR = 5.73, CI 1.66, 19.70), and those with higher hospital frailty risk score (ARS scale: AOR = 4.02, CI 1.74, 9.28) and higher number of medicines (ABC scale: AOR = 1.19, CI 1.09, 1.29, ACB scale: AOR = 1.23, CI 1.11, 1.35, ARS scale: AOR = 1.18, CI 1.09, 1.29, DBI scale: AOR = 1.33, CI 1.20, 1.49) had higher odds of anticholinergic exposure (Table 3). Compared to NZ European, Pacific patients had lower odds of anticholinergic exposure (DBI scale: AOR = 0.14, CI 0.03, 0.64). Similarly, increasing age was associated with lower odds of anticholinergic exposure (ACB scale: AOR = 0.36, CI 0.16, 0.79; DBI scale: AOR = 0.30, CI 0.14, 0.61). Further analysis by splitting the data into the oldest old (≥ 80 years) vs. younger old (65–79) did not show a significant difference between the two groups in anticholinergic exposure on admissions across all four scales. Similar results were observed at discharge, except for the DBI [crude’s odds ratio (COR) 0.57, 95%CI 0.31, 0.96) and ACB scales (COR 0.54, 95% CI 0.29, 0.99), where the ‘oldest old’ had lower odds of exposure. However, in multivariable logistic analysis, no significant differences were observed between the two groups across all scales, both on admission and at discharge.

**Table 3:** Factors associated with exposure to anticholinergics at discharge (*N* = *215*)

Variables	Exposure to anticholinergics at discharge			
	ACB scale AOR (95% CI)	ABC scale AOR (95% CI)	ARS scale AOR (95% CI)	DBI scale AOR (95% CI)
Age in years *	**0.36** ***(0.16–0.79)***	0.68 *(0.33–1.42)*	0.35 *(0.16–0.79)*	**0.30** ***(0.14–0.61)***
Sex *(ref male)*	1.25 *(0.62–2.54)*	0.66 *(0.33–1.32)*	1.43 *(0.66–3.08)*	1.00 *(0.53–1.91)*
Ethnicity (ref NZ European)				
Asian	0.78 *(0.22–2.72)*	0.90 *(0.27–3.00)*	0.48 *(0.11–2.13)*	0.72 *(0.24–2.20)*
Pacific	0.28 *(0.06–1.31)*	0.17 *(0.02–1.60)*	0.22 *(0.23–2.08)*	**0.14** ***(0.03–0.64)***
Māori	3.50 *(0.50-23.55)*	3.45 *(0.50–23.79)*	1.08 *(0.16–7.41)*	0.19 *(0.03–1.13)*
Number of medicines *	**1.23** ***(1.11–1.35)***	**1.19** ***(1.09–1.29)***	**1.18** ***(1.09–1.29)***	**1.33** ***(1.20–1.49)***
Deprivation Index *(ref least deprived)*				
Moderately deprived	1.74 *(0.82–3.70)*	1.19 *(0.57–2.51)*	1.12 *(0.50–2.52)*	1.64 *(0.83–3.22)*
Most deprived	0.74 *(0.29–1.86)*	1.86 *(0.73–4.76)*	1.52 *(0.56–4.14)*	1.08 *(0.45–2.58)*
CCI	1.01 *(0.80–1.27)*	1.02 *(0.82–1.26)*	0.79 *(0.61–1.03)*	0.96 *(0.79–1.16)*
Frailty Index *(ref non-frail)*				
*Pre-frail*	3.50*(0.86–14.23)*	0.80 *(0.16–4.05)*	1.40 *(0.27–7.21)*	**6.58** ***(1.71–25.32)***
*Frail*	**5.73** ***(1.66–19.70)***	2.52 *(0.67–9.52)*	1.43 *(0.35–5.80)*	2.88 *(0.97–8.51)*
HFRS *(ref low risk)*	0.91 *(0.42–1.95)*	1.14 *(0.54–2.39)*	**4.02** ***(1.74–9.28)***	1.64 *(0.81–3.28)*
Readmission history *(ref none)*	1.86 *(0.78–4.41)*	1.32 *(0.62–2.80)*	0.62 *(0.27–1.46)*	1.21 *(0.58–2.53)*
History of falls *(ref none)*	0.93 *(0.46–1.89)*	0.95 *(0.47–1.92)*	0.92 *(0.42–2.00)*	1.53 *(0.80–2.93)*
Length of stay *≥ median*	0.62 *(0.30–1.27)*	0.58 *(0.29–1.19)*	0.40 *(0.18–0.89)*	0.85 *(0.45–1.61)*

Bolded values = statistically significant (*p* < 0.05) higher odds of anticholinergic exposure, *AOR* = Adjusted Odds Ratio, *CI* = confidence interval, *ACB* = Anticholinergic Cognitive Burden, *ABC* = Anticholinergic Burden Classification, *ARS* = Anticholinergic Risk Scale, *DBI* = Drug Burden Index, ‘*ref’ =* Reference Group, *= continuous variable

## Discussion

Our study found 32–74% of older patients on admission, and 25–65% at discharge were prescribed at least one medicine with anticholinergic effect. Of these, 14–36% of the patients on admission and 8–30% at discharge had high anticholinergic burden. Overall, there was a reduction in the median anticholinergic burden from admission to discharge across the scales and that 12–33% of the patients had a decrease in their anticholinergic burden score at discharge. However, 32–85% of the patients had no change in their anticholinergic burden score from admission to discharge whilst 2.8–39.5% had an increase anticholinergic burden at discharge.

Our findings are consistent with local and international literature. Previous NZ studies reported that 3 in 4 older patients in acute setting (as per ADS scale) [27] and 1 in 3 in primary care (as per the DBI) are prescribed at least one anticholinergic medicine [17]. International studies that have examined anticholinergic burden in an acute setting reported conflicting results, with some reporting a reduction in the number of prescribed anticholinergic medicines from admission to discharge whilst others showing either no change or increased in anticholinergic burden from admission to discharge [27–30]. In an observational study of 549 older patients recruited from seven hospitals (four European countries), more than 1 in 5 patients who were not on anticholinergic medicines on admission were discharged with at least one anticholinergic medicine [28]. An Italian study of 1908 hospitalised older patients reported 38% of the patients had increased ACB score at discharge [30]. In a Spanish study of 200 patients in a geriatric unit [31], no significant change in the exposure to anticholinergics was found between admission and discharge.

In our study, we found a significant association between the number of medicines and the odds of anticholinergic burden both on admission and at discharge. A one unit increase in the number of medicines was associated with 1.2 to 1.3-fold increase in the odds of anticholinergic burden. As the number of medicines increases, the chance of taking an anticholinergic medicine and the cumulative anticholinergic burden increases. This needs critical consideration due to potential risk of inappropriate anticholinergic medicines use and associated adverse outcomes [32]. In community-dwelling older adults in New Zealand, anticholinergic agents such as tricyclic antidepressants and benzodiazepines account for 17% and 15% of inappropriately prescribed medicines, respectively [33]. Likewise, other classes of medicines with anticholinergic effects such as psychotropics significantly contribute to higher exposure to potentially inappropriate medicines in community-dwelling older adults in NZ [33].

We also found a high prevalence of frailty in our study population, with two-third of the patients being frail based on FI measure and more than one-third considered having a moderate risk as per hospital frailty risk score. The relationship between frailty and anticholinergic burden is intertwined. In our study, depending on the scale used, 77.8–86.2% of frail patients on admission and 75.6–87% of them at discharge had exposure to at least one anticholinergic medicine. Among patients with frailty, 12.4–40% on admission, and 7.9–34.8% at discharge had high anticholinergic exposure. A higher anticholinergic burden can worsen outcomes in frail patients, and frailty, can in turn, lowers individuals’ capability to handle adverse anticholinergic effects [34]. Thus, understanding individual’s frailty status and level of anticholinergic burden is essential to inform the risk-benefit assessment and optimise the use of anticholinergic medicines. In our study, both on admission and at discharge, the odds of anticholinergic burden were higher among frail patients than non-frail patients. However, this association was not consistently observed across all scales. A recent Australian study of 115 geriatric inpatients found that one third of severely frail patients had high anticholinergic burden and patients with higher anticholinergic burden were two times as likely to be severely frail [35]. The risk of adverse anticholinergic events such as falls among frail older patients with exposure to high anticholinergics is higher by about four times compared to non-frail patients [36]. While significant association between history of falls and exposure to anticholinergics was not observed in our study, a significant proportion (44%) of our study cohort had previous history of falls, of which, 13–32% on admission and 14–29% at discharge had exposure to anticholinergics bearing considerable risk of adverse cognitive and functional outcomes.

In our study, one in four patients had readmission history 28 days prior to current hospitalisation and patients with readmission history had almost five times higher odds of anticholinergic exposure on admission (shown in ACB scale only). This is broadly consistent with existing international literature [37–39]. Patients hospitalised with acute health care needs likely receive new treatment and/or changes to their pre-admission treatment regimen, which may be continued during hospitalisation to post-discharge. In a prospective study of 452 adult patients admitted to emergency surgical setting in the UK, moderate anticholinergic burden (measured by ACB scale) was significantly associated with 30-day readmission [37]. Likewise, a retrospective study of 3061 older inpatients in Taiwan reported that patients with ACB score of ≥ 2 had higher readmission within 6-month post discharge [39].

The median length of stay (LoS) in our study cohort was twenty days. No significant association between LoS and anticholinergic burden was observed across the scales except in the ARS scale which showed that patients with longer LoS had lower anticholinergic exposure than those with shorter LoS. One possible reason for this is that patients with longer LoS may have their medicines reviewed and anticholinergics deprescribed in the hospital. However, as our study cohorts were recruited from general medicine and geriatrics wards, whether the observed association between increased LoS and reduced anticholinergic exposure holds true across other specialities within the hospital needs investigation. It known that prescribing practices and deprescribing awareness vary between specialities. In a study of 33,360 of older inpatients in United Kingdom, Herrero-Zazo et al. [40] found a significant variation in anticholinergic burden (ARS score) between hospital specialties with lower anticholinergic burden scores in patients discharged from the Geriatric Medicine, and Trauma and Orthopaedics wards compared to other specialities.

In the present study, increasing age was associated with lower odds of anticholinergic exposure at discharge. The lower exposure to anticholinergics at discharge could be due to prioritisation of the older age group for medicine review to reduce medicines burden during hospitalisation. However, our analysis by splitting the data into the oldest old (≥ 80 years) vs. younger old (65–79), did not show significant difference in the anticholinergic exposure between the two groups. Hence, further research is required if hospital-based high risk patient prioritisation for targeted interventions such as deprescribing can reduce anticholinergic burden. We also found lower anticholinergic exposure (DBI scale) at discharge among pacific patients compared to NZ Europeans. Our finding aligns with previous community-based NZ studies which reported lower DBI exposure among Pacific and Māori compared to NZ Europeans [17, 18]. These findings signal differences in anticholinergics prescribing exit in different ethnic groups in NZ. However, further research is needed to understand if inequities in prescribing practices or differences in medical conditions among different ethnic groups is a driving factor.

### Limitations

There are several limitations to our study. The participants were recruited from general medicine and geriatrics wards, and older inpatient without cognitive impairment and thus, the findings cannot be generalised beyond the population studied. Further research is needed to gain insights into exposure to anticholinergics in other specialities within the hospital or other hospitals across the country. Both Māori and Pacific patients were underrepresented in our study which could be attributed to our inclusion criteria. We used ≥ 65 years as cut-off to define older adults irrespective of their ethnicity. As there is a significant difference in life expectancy among ethnic groups in NZ, using ≥ 50 years cut-offs for Māori and Pacific patients may have improved representation of both groups in our study. Although several scales exist to measure the cumulative effects of anticholinergic medicines, there is no universally accepted gold standard method to assess the anticholinergic burden. Due to heterogeneity between the scales in their underlying assumptions for quantifying anticholinergic burden, list of medicines included and rating the medicines [41], it was not possible to pool the data from each scale as a single absolute number. We haven’t categorised classes of medicines contributing to anticholinergic burden in this study and thus, future research should explore the most common contributing classes of medicines to anticholinergic burden to inform decision making. Furthermore, causality between identified factors and anticholinergic exposure cannot be determined. However, given high proportion of our study cohort were discharged with anticholinergic medicines and that anticholinergic exposure in acute setting is a risk factor for hospital readmissions, using anticholinergic burden scales alongside existing clinical decision-support system might present better opportunity to reduce exposure to these medicines in the hospital and potential adverse outcomes post-discharge.

Despite these limitations, we used a comprehensive approach to evaluate anticholinergic burden using multiple scales to provide insights into exposure to anticholinergic medicines in acute setting in NZ. The findings can inform clinical practice and future research on implementation of targeted interventions to reduce anticholinergic exposure in an acute setting particularly, in the most vulnerable group such as patients with frailty and high anticholinergic burden.

## Conclusion

A reduction in the anticholinergic burden from admission to discharge was observed in the study population yet, one-third of the study cohort were discharged with medicines with high anticholinergic effects. Enhancing hospital prescribers’ and pharmacists’ awareness about anticholinergic burden and targeted interventions such as in-hospital deprescribing are needed to reduce high anticholinergic exposure in acute setting.

## Electronic supplementary material

Below is the link to the electronic supplementary material.

Supplementary Material 1

## Data Availability

The datasets used and /or analyzed during this study are not publicly available due to confidentiality of patient information and ethical restrictions but are available in summary form from the corresponding author upon reasonable request.

## Abbreviations

ABC: Anticholinergic Burden Classification
ACB: Anticholinergic Cognitive Burden
AOR: Adjusted odds ratio
ARS: Anticholinergic Risk Scale
CCI: Charlson comorbidity index
CIs: Confidence intervals
DBI: Drug Burden Index
FI: Frailty index
HFRS: Hospital frailty risk score
IQR: Interquartile range
ICD-10-AM: International Classification of Diseases 10th Revision-Australian Modification
SD: Standard deviation
