# Supplementary material for: Anticholinergic burden and frailty in older inpatients: insights from analysis of admission and discharge medicines using four anticholinergic scales
Source: BMC Geriatr. 2024 Dec 20;24:1022. doi: 10.1186/s12877-024-05394-3 (PMC11660849; doi:10.1186/s12877-024-05394-3)
Supplement: Supplementary file 1 — Supplementary Material 1 [file 12877_2024_5394_MOESM1_ESM.docx]

**List of deficits used to quantify Hospital Frailty Risk Score (HFRS)**

| **Codes** | **Definition** | **Point awarded** |
| --- | --- | --- |
| **F00** | Dementia in Alzheimer | 7·1 |
| **G81** | Hemiplegia | 4·4 |
| **G30** | Alzheimer's disease | 4·0 |
| **I69** | Sequelae of cerebrovascular disease (secondary codes) | 3·7 |
| **R29** | Other symptoms and signs involving the nervous and musculoskeletal systems (R29·6 Tendency to fall) | 3·6 |
| **N39** | Other disorders of urinary system (includes urinary tract infection and urinary incontinence) | 3·2 |
| **F05** | Delirium, not induced by alcohol and other psychoactive substances | 3·2 |
| **W19** | Unspecified fall | 3·2 |
| **S00** | Superficial injury of head | 3·2 |
| **R31** | Unspecified hematuria | 3·0 |
| **B96** | Other bacterial agents as the cause of diseases classified to other chapters (secondary code) | 2·9 |
| **R41** | Other symptoms and signs involving cognitive functions and awareness | 2·7 |
| **R26** | Abnormalities of gait and mobility | 2·6 |
| **I67** | Other cerebrovascular diseases | 2·6 |
| **R56** | Convulsions, not elsewhere classified | 2·6 |
| **R40** | Somnolence, stupor and coma | 2·5 |
| **T83** | Complications of genitourinary prosthetic devices, implants and grafts | 2·4 |
| **S06** | Intracranial injury | 2·4 |
| **S42** | Fracture of shoulder and upper arm | 2·3 |
| **E87** | Other disorders of fluid, electrolyte and acid-base balance | 2·3 |
| **M25** | Other joint disorders, not elsewhere classified | 2·3 |
| **E86** | Volume depletion | 2·3 |
| **R54** | Senility | 2·2 |
| **F03** | Unspecified dementia | 2·1 |
| **W18** | Other fall on same level | 2·1 |
| **Z75** | Problems related to medical facilities and other health care | 2·0 |
| **F01** | Vascular dementia | 2·0 |

**List of deficits used to quantify Frailty Index(FI)**

|  | **Deficits** |
| --- | --- |
| 1 | **Arthritis** |
| 2 | **Atrial Fibrillation** |
| 3 | **Bowel incontinence** |
| 4 | **Cancer** |
| 5 | **Coronary Heart Disease** |
| 6 | **Diabetes** |
| 7 | **Depression** |
| 8 | **Psychotic disorders** |
| 9 | **Heart Failure** |
| 10 | **Hypertension** |
| 11 | **Stroke and TIA** |
| 12 | **Hypotension/Syncope** |
| 13 | **Renal disease** |
| 14 | **Fracture***:* *hip fracture, fragility fracture* |
| 15 | **Respiratory disease** |
| 16 | **Skin Ulcer**: *pressure sore, dressing of ulcer* |
| 17 | **Urinary system disease**: *Urinary incontinence, Urinary tract infection* |
| 18 | **Memory and cognitive problems**: *Dementia* |
| 19 | **Polypharmacy** |
| 20 | **Foot problems***: swollen foot, foot pain* |
| 21 | **Sleep disturbance**: *Insomnia* |
| 22 | **Weight loss and anorexia** |
| 23 | **Malnutrition** |
| 24 | **Mobility problems:** *activity limitation****,*** impaired *mobility, needs/use walking aid* |
| 25 | **Requirement for care**: *Lives in care home/ has a carer* |
| 26 | **Social vulnerability**: *No spouse, lives alone/no help available* |
